# Leveraging health care settings to strengthen trust in light-touch benefit outreach

**DOI:** 10.1073/pnas.2537008123

**Published:** 2026-05-18

**Authors:** Matthew C. Guido, Madeline P. Maier, Alister F. Martin

**Affiliations:** ^a^Link Health, A Healthier Democracy, Boston, MA 02111; ^b^Department of Emergency Medicine, Massachusetts General Hospital, Boston, MA 02114

We commend Linos et al. for offering actionable evidence on how outreach intensity, framing, and targeting shape public benefit take-up at scale. Their findings show that simple, low-cost nudges conducted by a government agency, such as text messages, emails, and recorded voice messages, can increase enrollment nearly as effectively as higher-touch outreach efforts (1). Yet even behaviorally informed nudges may falter when delivered by unfamiliar or mistrusted senders, or when individuals encounter administrative complexity during enrollment. Messenger trust may therefore be a critical, underexamined determinant of whether light-touch outreach interventions succeed, particularly among underrepresented populations and those facing language or administrative barriers.

The 2021 Child Tax Credit (CTC) expansion illustrates both the promise and limits of light-touch outreach. As Linos et al. note, California’s campaign achieved modest, cost-effective enrollment gains through reminder messaging. Still, millions of eligible families, disproportionately immigrant and non-English-speaking households, did not receive benefits. Local evaluations found higher CTC uptake when messages came from trusted, bilingual community organizations rather than impersonal federal notices (2). Similarly, Code for America’s GetCTC.org initiative increased enrollment when outreach was paired with community-based assistance (3). This suggests light-touch strategies are most effective when paired with trusted intermediaries capable of supporting navigation of multistep enrollment processes.

Health care settings may represent an especially scalable and trusted intermediary for engagement across different benefit programs and through multiple steps of the enrollment process. Primary care clinics and emergency departments routinely serve populations eligible for the Supplemental Nutrition Assistance Program (SNAP), the Special Supplemental Nutrition Program for Women, Infants, and Children (WIC), Medicaid, and utility assistance. Clinicians and local health institutions consistently rank among the most trusted sources of information in the United States, and participation in social safety net programs is a core mechanism for addressing health-related social needs by providing financial assistance, nutritional assistance, housing support and access to health care (4–6). Embedding benefit outreach within care delivery may amplify the impact of low-intensity nudges while mitigating distrust and administrative attrition.

Emerging evidence supports this approach. A randomized trial in two emergency departments found that postdischarge automated text messages referring eligible patients to a benefits navigator, compared with paper discharge materials alone, resulted in 18% enrolling in at least one benefit vs. 0% in the control group (7). Clinic-based models leveraging multilingual text messaging or brief, in-person navigation have similarly translated outreach into durable uptake. In one citywide initiative across federally qualified health centers, nearly 70,000 patients received text messages from their clinic, resulting in more than 1,400 successful enrollments in the Lifeline program, with substantial participation among Spanish-speaking households (8). In-clinic navigation by the Link Health initiative (with which the authors are affiliated) has used brief eligibility screening and real-time application support to connect thousands of patients to federal programs, generating over $2.8 million in benefits (9, 10). These place-based strategies leverage existing patient relationships, reduce administrative friction, and reinforce the relevance of benefit programs within routine care.

As governments refine outreach strategies to support public benefit delivery, partnerships with trusted health care institutions offer a practical path to scale. Such collaborations pair behavioral efficiency with credible messengers and real-time enrollment support to embed trust directly into outreach design.
